# Stress Response Pathways in Ameloblasts: Implications for Amelogenesis and Dental Fluorosis

**DOI:** 10.3390/cells1030631

**Published:** 2012-08-30

**Authors:** Megan L. Sierant, John D. Bartlett

**Affiliations:** 1 Department of Mineralized Tissue Biology, Forsyth Institute, 245 First Str., Cambridge, MA 02142, USA; Email: msierant@forsyth.org; 2 Harvard School of Dental Medicine, 188 Longwood Ave, Boston, MA 02115, USA

**Keywords:** enamel, amelogenesis, fluorosis, ameloblast, endoplasmic reticulum stress, unfolded protein response

## Abstract

Human enamel development of the permanent teeth takes place during childhood and stresses encountered during this period can have lasting effects on the appearance and structural integrity of the enamel. One of the most common examples of this is the development of dental fluorosis after childhood exposure to excess fluoride, an elemental agent used to increase enamel hardness and prevent dental caries. Currently the molecular mechanism responsible for dental fluorosis remains unknown; however, recent work suggests dental fluorosis may be the result of activated stress response pathways in ameloblasts during the development of permanent teeth. Using fluorosis as an example, the role of stress response pathways during enamel maturation is discussed.

## 1. Introduction

Dental enamel is produced by specialized epithelial-derived cells known as ameloblasts, which are a layer of tall columnar polarized cells, and is the product of ameloblast progression through the various stages of their life cycle. In order to produce enamel, ameloblasts must synthesize and secrete large amounts of different proteins. This makes enamel especially susceptible to perturbations in ameloblast protein synthesis, both from internal and external sources. Perturbations in protein synthesis and secretion from trauma or illness can be observed visually in the teeth of affected individuals as unusually prominent Striae of Retzius, which are visible manifestations of the ameloblast growth cycle analogous to the growth rings of a tree [1]. Both fever lines on the permanent teeth, caused by high fever during childhood, and the neonatal line on the deciduous teeth, caused by the trauma associated with being born, are two very common examples of bodily stressors causing interruptions in enamel development [1].

Fluoride is used to increase enamel hardness and prevent the formation of caries by incorporation into the hydroxyapatite (HA) crystals that make up the bulk of dental enamel. Fluoride is not produced by the body and so must be provided as a supplement, either from community water programs or in the form of gels, coatings, or rinses supplied by dental professionals. The dose required for beneficial effects of fluoride is quite low with community water guidelines stating that 0.7 parts per million (ppm) is sufficient [2]. At doses higher than 0.7 ppm, the risk of the development of dental or skeletal fluorosis increases and the detriments begin to outweigh the benefits. Dental fluorosis is the result of excess fluoride during enamel development, which for the permanent teeth occurs in childhood between the ages of 2 to 8 years old, and affects roughly one quarter of the American population [3]. Once enamel has fully matured, the risk of developing dental fluorosis is eliminated. Dental fluorosis produces areas of weakened enamel that appear as opaque white spots or lines, with severity proportional to the affected surface area. In the most severe cases, enamel can become discolored and brittle, leading to chipping. These spots or lines are areas of weakened enamel that have higher than normal protein content [4,5,6] and it is the increased protein content in these areas that results in the observed enamel weakness of fluorosed enamel. Current work suggests the involvement of the protein synthesis machinery in the development of dental fluorosis [7,8,9] and this review uses fluorosis to illustrate the importance of the stress response pathways on protein production during amelogenesis.

## 2. Enamel Development

In order to better understand the role of protein synthesis during amelogenesis, a brief review of ameloblast function and enamel development follows. Amelogenesis, or enamel development, occurs over five stages but two stages are arguably the most important: the secretory stage and the maturation stage (reviewed in [10]). In the secretory stage, enamel proteins are secreted, which are required to support and organize the nascent HA crystals as the enamel layer grows to its full thickness [10]. In the maturation stage, the matrix proteins are degraded by a stage-specific protease and the fragments resorbed, allowing for further HA precipitation and crystal growth as the enamel matures into its final hardened form [10].

### 2.1. The Secretory Stage

In the secretory stage, the enamel reaches full thickness but is composed of a soft, cheese-like substance easily separated from the dentin by mechanical techniques. This is due to the high levels of protein still found in the enamel matrix (reviewed in [11]). Due to the high amounts of proteins produced and secreted, secretory ameloblasts are tall with well developed endoplasmic reticulum (ER) and golgi bodies [12]. Ameloblasts also develop a specialized conical structure on the apical surface known as a Tomes' process, an easily recognizable landmark from which the enamel rod will grow and various proteins required for enamel formation are secreted. These proteins include amelogenin (AMELX), ameloblastin (AMBN), enamelin (ENAM), and the secretory-staged protease enamelysin or matrix metalloproteinase 20 (MMP20). AMELX, AMBN, and ENAM are scaffold proteins required to support rod formation and HA crystallization. These proteins are broken down by MMP20 as they are secreted into the enamel matrix, meaning the full-length proteins are located closest to the Tomes’ process and the smallest fragments closer to the dentino-enamel junction, and this breakdown is necessary for enamel development [13,14,15] as well as HA crystal maturation [16,17]. Once the enamel has reached full thickness, ameloblasts transition to the maturation stage [11].

### 2.2. The Maturation Stage

By the beginning of the maturation stage, the enamel has reached full thickness [11] and, in rodents, has developed the characteristic decussating enamel rod pattern; however, the enamel has not yet hardened [11]. During the maturation stage, the remaining proteins in the enamel matrix will be degraded by the stage-specific serine protease kallikrein-related peptidase-4 (KLK4), also known as enamel matrix serine protease 1 (EMSP1), and the protein fragments generated are absorbed and further degraded by the ameloblasts [18,19]. This decreases the amount of enamel matrix protein from approximately 30% by weight to roughly 1% by the end of maturation stage. While the enamel matrix is undergoing proteolysis, HA precipitates increasing the density of the enamel rods and filling the spaces created during matrix degradation [11]. Rat ameloblasts undergo cyclic oscillations during this stage, with one cycle taking approximately 8 hours [20]. For roughly 50% of a cycle ameloblasts exist in their ruffle-ended (RE) form, and spend the next 25% in their smooth-ended (SE) form; the remaining 25% of a cycle is spent transitioning between these two forms [20] with the transition from RE to SE taking more time than the SE to RE transition [21]. Rat incisor ameloblasts will undergo the RE-SE cycle approximately 3 times per day and at least 45 times by the end of the maturation stage [20].

RE ameloblasts are readily identified by large invaginations on their apical surface, which are believed to simultaneously secrete the enamel protease KLK4, absorb degraded enamel matrix for intracellular breakdown [22,23,24,25], and secrete large amounts of calcium ions into the enamel matrix to facilitate HA precipitation [26]. Due to the efflux of calcium and phosphate ions, which causes an increase in HA precipitation and subsequently the release of hydrogen ions, the pH of the enamel matrix associated with RE ameloblasts drops [27]. Precipitation of HA releases 7–14 moles of hydrogen ions per mole of HA, which acidifies the enamel matrix, and both RE and SE ameloblasts secrete bicarbonate to neutralize the enamel matrix. SE ameloblasts also provide a resting phase for the ameloblast, allowing time to process the degraded enamel matrix and reset for the next RE phase [11]. These modulations occur in waves around the developing tooth though the signal to switch phases remains unknown [11].

### 2.3. Enamel Defects

To illustrate the importance of protein secretion during the development of healthy enamel, defects associated with secretion or production of any of these important proteins results in malformed enamel in both mice and humans (reviewed in [28]). For example, AMELX-deficient mice display disorganized (lacks the characteristic decussating rod pattern), thin enamel [29]. In humans, there are currently 16 different mutations associated with *Amelx* which result in X-linked amelogenesis imperfecta (AI) [28,30]. AI is a term for a collection of non-syndromic hereditary enamel defects which vary in severity and appearance. *Ambn*^−/−^ mice have a severe phenotype presenting severely malformed, very thin enamel [31]. No AI-causing mutation in the AMBN gene has been identified in humans to date [32]. *Enam^−/−^* mice lack true enamel [33] and in humans mutations result in autosomal dominant AI [28], in which only one allele has to be affected in order for AI to develop. Humans with autosomal dominant AI have extremely discolored, small teeth with very thin, weak enamel [34]. Afflicted individuals may also display prominent banding of malformed enamel [35] or caries-like lesions [36].

Mutations affecting the enamel matrix proteases, MMP20 and KLK4, can also produce the AI enamel phenotype in an autosomal recessive manner [32,37,38]. A complete loss of MMP20 expression results in thin, weak, disorganized enamel in mice [13]. Aberrant MMP20 in humans, via mutations producing either a premature stop codon, disrupted active site, or inactive splice variant, also show enamel defects [39,40,41,42]. Affected individuals present with weakened, discolored enamel susceptible to chipping though, unlike the murine phenotype, enamel is often full thickness [39,40]. To date only one AI-causing KLK4 mutation has been identified in humans, producing a truncated protein, lacking an important component of the active site, which produces weak immature enamel of normal thickness [43]. This is due to a lack of mineralization during the maturation phase and, subsequently, an increased enamel protein content [43]. In mice, *Klk4*-knockout produces enamel of normal thickness showing the decussating rod pattern; however, this enamel is quite brittle, breaking close to the dentino-enamel junction (DEJ) [38]. Additionally, the individual HA crystallites, which normally fuse to become a single enamel rod, fail to fuse and can be individually isolated from neighboring crystallites, indicating KLK4 is required for proper rod formation and enamel maturation [38]. AI resulting from mutation of these two proteases show that both enzymes are required for proper amelogenesis in humans as well as rodents [13,39,40,41,42,43].

In addition to the canonical enamel proteins, defects in members of other cellular processes can also produce a dysplastic enamel phenotype. AI has also been linked to mutations in two members of the family with sequence similarity (FAM) gene family, FAM83H and FAM20A, as well as WD repeat containing domain 72 (WDR72) [44,45,46]. Despite the linkage of these genes with the AI phenotype, their function in enamel development remains unclear [44,45,46]. Furthermore, mutations in the cystic fibrosis transporter gene (*Cftr*), in addition to causing Cystic Fibrosis, produces thin malformed enamel in both mice and humans [47,48,49], underscoring the importance of ion management during enamel maturation. The CFTR-related enamel phenotype does not fall under the AI "umbrella" and therefore is not included in a list of known AI-causing genes despite its influence on enamel development [50]. Though several AI-causing genes have been identified, there are still a number of cases in which no mutation or genetic defect has been identified indicating that there is still much work to done in this field. The variety, and in some cases severity, of enamel defects associated with proper protein production underscores its importance in ameloblasts during enamel development.

## 3. Fluoride and Ameloblasts

Fluoride is a widely used supplement that increases enamel hardness and reduces the incidence of dental caries [51]. In contrast to this protective effect, excess fluoride during childhood (ages 2–8; when the enamel of the permanent teeth is developing) has the opposite outcome, weakening enamel and resulting in areas of increased opacity (reviewed in [52]). In the most severe cases of dental fluorosis, the enamel can become discolored and chip resulting in jagged, uneven tooth surfaces [52]. In addition to being a cosmetic issue, fluorosed enamel is weaker [5,6], more porous [53,54], and contains higher levels of protein than healthy enamel [4,54] so dental fluorosis also has physical effects.

### 3.1. Fluoride does not Affect the Activity of MMP20 or KLK4

Fluorosed enamel contains more protein than normal enamel leading researchers to speculate that decreased enamel protease function in the presence of fluoride was to blame [55]. However, in the case of both recombinant MMP20 and KLK4 as well as pooled enamel organ protein extracts (containing both MMP20 and KLK4), fluoride has no effect on the activity of these two enzymes, ruling out protease inhibition as a potential mechanism [56,57]. So, while fluorosed enamel contains increased protein levels, it is not the direct result of decreased protease activity.

### 3.2. Fluoride Causes Oxidative Stress

Fluoride increases oxidative stress by reducing the activity of antioxidant enzymes [58,59,60] resulting in the accumulation of reactive oxygen species (ROS), which negatively affect a variety of structures and processes in the cell. Increases in ROS levels can activate the general stress response protein heat shock protein 90 (HSP 90) which arrests protein synthesis by activating the eukaryotic initiation factor 2 (eIF2) kinase heme regulated inhibitor (HRI) [61,62,63]. Treatment with antioxidant compounds such as selenium, lycopene, or α-tocopherol (vitamin E) can ameliorate the increase in ROS levels caused by excess fluoride [60,64,65,66,67,68] but whether these compounds are able to restore enamel hardness remains to be seen.

Fluoride also induces expression of genes associated with oxidative stress [69]. In osteoblasts, fluoride causes an increase in the nuclear factor erythroid 2-related factor 2 (NRF2) [8] but it is unknown whether this occurs in ameloblasts. NRF2 is a cytoprotective transcription factor involved in the response to increased ROS levels (reviewed in [70]) as well as in hematopoietic stem cell maintenance [71]. NRF2 is also required for iron deposition on murine incisors, via regulation of ferritin levels, and deficiencies in NRF2 caused premature ameloblast atrophy during late maturation stage [72]. Though there was no difference in enamel hardness between WT and *Nrf2*^−/−^ mice, the enamel from null mice was more sensitive to acid, leaching greater amounts of calcium than control teeth [72]. This could result from the absence of the iron-based enamel top-layer [72], which gives murine incisors their characteristic orange coloring [73]. Fluorosed murine enamel also lacks the iron-based coating [74], suggesting fluoride may inhibit NRF2-mediated iron deposition in exposed animals.

As previously mentioned, oxidative stress can decrease protein synthesis through the HSP90-eIF2 mechanism but high levels of ROS can also affect proteins in other ways. One way oxidative stress can also affect protein function is through a direct or indirect post-translational modification of amino acid side chains known as carbonylation. Carbonylation can both positively and negatively affect protein function (reviewed in [75]), depending on the protein, or can increase proteosomal degradation. NRF2 has been shown to be upregulated in cells containing high levels of carbonylated proteins, via increased dissociation from its inhibitor KEAP1 [76]. Decreased protein synthesis via HSP90 and increased protein degradation through protein carbonylation could result in decreased levels of both MMP20 and KLK4, which would lead to increased levels of enamel matrix proteins and could be a potential mechanism for fluorosis.

### 3.3. Fluoride Induces ER Stress and eIF2 Phosphorylation

The unfolded protein response (UPR) has evolved to sense the accumulation of misfolded proteins and halt protein synthesis until the misfolded proteins are refolded or degraded (reviewed in Hetz [77]). UPR is a three-pronged pathway which will result in the increase of chaperone and ER-assisted protein degradation (ERAD) proteins while preventing global protein translation, via phosphorylation of eIF2α and mRNA degradation [77]. Sensors of the UPR include activating transcription factor 6 (ATF6), inositol-requiring protein 1α (IRE1α), and protein kinase RNA-activated ER kinase (PERK) [77]. All three sensors are transmembrane proteins spanning the ER membrane with the sensor domains projecting into the ER lumen and the signaling domains projecting into the cytosol [77].

Fluoride has been shown to activate PERK in osteoblasts [8] and causes ER stress in ameloblasts, which decreases protein secretion [9,78]. Additionally, PERK is capable of activating NRF2 [79] and the two play an important role during redox stress (reviewed in Cullinan and Diehl [80]). Given that fluoride decreases synthesis and secretion of the enamel proteases by decreasing mRNA translation [7,81], the involvement of the UPR in dental fluorosis seems likely.

Treatment of ameloblast lineage cells with fluoride induces eIF2α phosphorylation in vitro, which is increased under mildly acidic conditions, but the kinase responsible is currently unknown [7,82]. In addition to PERK, there are three other known kinases that act on eIF2α, the previously mentioned HRI, protein kinase RNA-activated (PKR), and glucose control nonrepressed 2 (GCN2) (reviewed in [83]). Each kinase responds to a particular set of stimuli: HRI phosphorylates eIF2α in response to heme depletion, arsenite exposure, oxidative stress, and heat shock; PKR is activated in response to increased interferon levels from viral infection; and GCN2 responds to nutrient limitation such as a depleted pool of amino acids [83]. In addition to PERK, HRI is another likely candidate for playing a role in response to fluoride. HRI is activated by oxidative stress induced by the environmental toxin arsenite [61,84], possibly via an intermediary [85], as well as oxidative stress induced by other compounds [62,63]. Interestingly, treatment with both fluoride and arsenite simultaneously does not induce a synergistic effect which implies that both fluoride and arsenite activate the same biochemical pathways [86]. These data point to HRI as being the activated eIF2α kinase; however, it is also possible that both PERK and HRI phosphorylate eIF2α in concert during exposure to fluoride, as both kinases act on the same pool of eIF2α and both have been shown to play a role during oxidative stress.

Phosphorylation of eIF2α does not halt all protein synthesis, mRNA species possessing alternate upstream open reading frames (uORFs) may still be translated [87]. Phosphorylation results in depleted pools of eIF2-Met tRNAi which can cause the regular ORF to be skipped in favor of the uORF in certain mRNA species, such as activating transcription factor 4 (ATF4) [88]. Translation of ATF4 is increased when eIF2 is phosphorylated [88], which in turn increases transcription of downstream UPR genes, aiding the cell in reestablishing homeostasis. ATF4 and NRF2 have been shown to interact and play a role in the activation of ROS resolving proteins [80,89]; however, to date fluoride has not been shown to increase ATF4 levels.

## 4. The Acid Hypothesis

As mentioned above, normal enamel development includes oscillations in pH during the maturation stage, cycling between neutral (pH 7.4) and mildly acidic (pH < 6.0) [90], which results from proton release during HA precipitation [91,92]. According to the Henderson-Hasselbalch equation, approximately 25 times more hydrogen fluoride (HF) is present at pH 6.0 compared to pH 7.4. Fluoride ions are unable to enter the cell whereas HF is readily able to cross the plasma membrane meaning that, under acidic conditions, there is more HF available to cross the cell membrane [93]. Additionally, studies show that a pH gradient is required in order to drive cellular fluoride absorption [94]. This makes maturation stage ameloblasts uniquely sensitive to fluoride toxicity, which has been observed experimentally [82,95,96,97,98]. Greater levels of dental fluorosis were observed in rats under acidic conditions when compared to basic or neutral conditions [99,100]. Additionally, induction of severe acidosis in rats produces an enamel phenotype similar to dental fluorosis [101]. In this study, Whitford and Agmar-Mansson observed elevated enamel fluoride levels in acidotic rats compared to control [101], which may result from increased fluoride mobilization from mineralized tissue under acidic conditions [102]. Taken together, these data illustrate the importance of pH in influencing the effects of fluoride.

**Figure 1:**
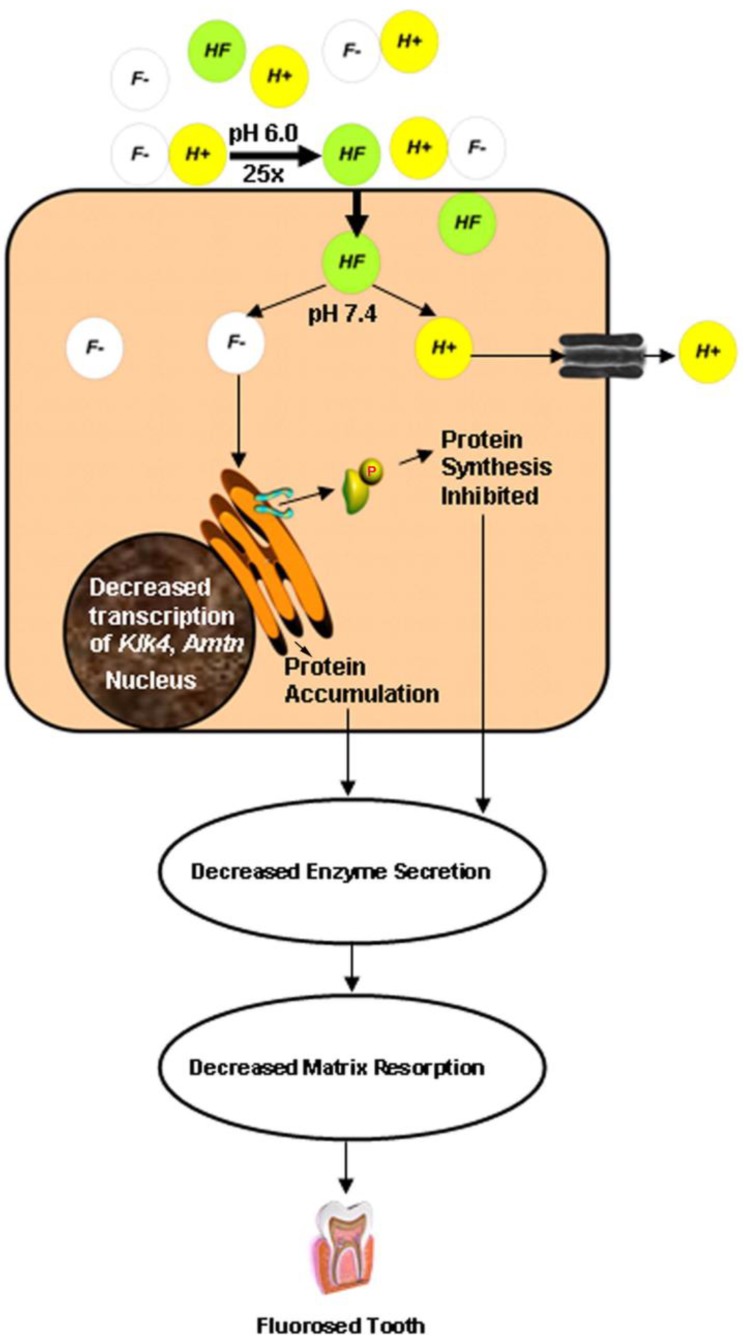
Schematic showing a postulated mechanism for maturation stage ameloblast sensitivity to fluoride. During the maturation stage, massive precipitation of hydroxyapatite occurs, releasing H^+^ ions. F^-^ can reversibly associate with H^+^ ions to form HF. Approximately 25-fold more HF is formed at pH 6.0 as compared to pH 7.4. HF diffuses into the cell more easily than F^-^ and flows down a steep concentration gradient from the acidic maturation stage enamel matrix into the neutral cytosol of the ameloblast. The neutral pH inside the cell causes reversion of HF to F^-^. Excess F^-^ within the cell interferes with ER homoestasis that may result in the dimerization and phosphorylation of PERK and its substrate, eIF2α. Consequently, protein synthesis is attenuated. ER stress can also lead to increased degradation of transcripts encoding secreted proteins such as KLK4. Collectively, decreased secretion of matrix-degrading enzymes such as KLK4 can lead to delayed resorption of enamel matrix proteins, resulting in the higher protein content observed in fluorosed enamel. ER, endoplasmic reticulum. Reproduced with permission from [82].

In cultured ameloblast-derived cells treated with fluoride, proteins associated with cellular stress (JNK and c-jun) were more readily phosphorylated under acidic conditions compared to neutral conditions [82]. These cells also showed signs of increased ER stress, including phosphorylation of eIF2α, under these same conditions which contributed to a general decrease in protein secretion in these cells [82]. Decreased protein production in maturation stage ameloblasts could lead to decreased production and secretion of KLK4, which would result in decreased enamel matrix breakdown and lead to increased protein content characteristic of fluorosed enamel. In fact, decreased protein production has already been shown in cultured ameloblasts exposed to fluoride [7,9,81] as well as in enamel isolated from fluoride-treated rats [98]. Maturation-staged ameloblasts also undergo fewer RE-SE modulation cycles in the presence of fluoride [97], fewer cycles could correspond to less time for matrix resorption further contributing to the increased protein levels observed in fluorosed enamel. This Acid Hypothesis states that the mildly acidic enamel matrix drives HF down a concentration gradient into the cytosol of maturation-staged ameloblasts where it dissociates. This both strengthens the HF concentration gradient and releases highly reactive fluorine ions into the cytoplasm leading to phosphorylation of eIF2α thereby decreasing overall protein production, including the secretion of the maturation stage protease KLK4. Consequently, there is less break down of the enamel matrix and this leads to increased levels of matrix proteins (Figure 1). DenBesten showed increased protein levels in fluorosed maturation-staged enamel and postulated it was the result of decreased protein removal [103]; these observations are consistent with our hypothesis. Current work by the Bartlett lab is focussed on validating this hypothesis.

## 5. Conclusions

The importance of producing correctly folded and functional enamel proteins is demonstrated by the wide variety and abundance of enamel defects that are traceable back to a single aberrant protein (AI) or to disrupted protein synthesis (neonatal line). Likewise, understanding how particular compounds affect proper ameloblast function is equally important for two reasons: Firstly, for providing additional insight into the mechanism of enamel development, and secondly for developing methods to avoid the development of, or for the treatment of, existing enamel formation disorders.
